# US Federal Travel Restrictions for Persons with Higher-Risk Exposures to Communicable Diseases of Public Health Concern

**DOI:** 10.3201/eid2313.170386

**Published:** 2017-12

**Authors:** Laura A. Vonnahme, M. Robynne Jungerman, Reena K. Gulati, Petra Illig, Francisco Alvarado-Ramy

**Affiliations:** Centers for Disease Control and Prevention, Atlanta, Georgia, USA (L.A. Vonnahme, P. Illig, F. Alvarado-Ramy);; Centers for Disease Control and Prevention, Reston, Virginia, USA (M.R. Jungerman);; Centers for Disease Control and Prevention, Seattle, Washington, USA (R.K. Gulati)

**Keywords:** travel, restrictions, public health travel restrictions, global health security, communicable diseases, epidemics, Ebola, Middle East respiratory syndrome coronavirus, MERS-CoV, viruses, Lassa fever, exposures, do not board, zoonoses, United States

## Abstract

Published guidance recommends controlled movement for persons with higher-risk exposures (HREs) to communicable diseases of public health concern; US federal public health travel restrictions (PHTRs) might be implemented to enforce these measures. We describe persons eligible for and placed on PHTRs because of HREs during 2014–2016. There were 160 persons placed on PHTRs: 142 (89%) involved exposure to Ebola virus, 16 (10%) to Lassa fever virus, and 2 (1%) to Middle East respiratory syndrome coronavirus. Most (90%) HREs were related to an epidemic. No persons attempted to travel; all persons had PHTRs lifted after completion of a maximum disease-specific incubation period or a revised exposure risk classification. PHTR enforced controlled movement and removed risk for disease transmission among travelers who had contacts who refused to comply with public health recommendations. PHTRs are mechanisms to mitigate spread of communicable diseases and might be critical in enhancing health security during epidemics.

In August 2014, the World Health Organization declared the Ebola virus disease outbreak in West Africa a public health emergency of international concern. In response to this outbreak, the Centers for Disease Control and Prevention (CDC) published Interim U.S. Guidance for Monitoring and Movement of Persons with Potential Ebola Virus Exposure, known as the Monitoring and Movement Guidance (*1*). This guidance recommended controlled movement, which was defined as limitation of long-distance travel by commercial means, for persons with higher-risk exposures (HREs), which were defined as having had a high-risk exposure to Ebola virus on the basis of epidemiologic risk factors or close contact with a person with symptomatic Ebola for a prolonged period who was not using appropriate personal protective equipment (*1**,**2*). In addition, in March 2015, CDC published revised criteria for use of federal public health travel restrictions (PHTRs) in the Federal Register so that these tools could be used to prevent travel of persons exposed to a communicable disease of public health concern and to support enhanced public health response to communicable disease outbreaks (Table 1) (*3*).

**Table 1:** Criteria for placement and removal from federal public health travel restrictions, March 2015*

Criteria for placement	**Criteria for removal**
Be known or likely infectious with, or exposed to, a communicable disease that poses a public health threat	Proven noninfectiousness or no longer being at risk for becoming infectious (by documented laboratory confirmation, lapse of known period of infectiousness, or lapse of incubation period without development of symptoms)
AND meet 1 of the following 3 criteria	
1) Be unaware of diagnosis, noncompliant with public health recommendations, or unable to be located	
OR	
2) be at risk for traveling on a commercial flight, or internationally by any means	
OR	
3) travel restrictions are warranted to respond effectively to a communicable disease outbreak or to enforce a federal or local public health order.	

*Criteria were obtained from the Centers for Disease Control and Prevention (*3*).

CDC uses federal PHTRs to protect the traveling public by preventing commercial air travel or other means of international travel across US borders of persons with a communicable disease or at risk for development of a disease that poses a public health threat (*3**,**4*). Federal mechanisms used to implement travel restrictions include the public health do not board (DNB) and Public Health Lookout lists (*5**,**6*). The DNB tool was developed in 2007 to prevent persons who met criteria (Table 1) from boarding commercial flights of any duration that have departures to or from the United States (*5**,**6*). A Public Health Lookout list is issued to complement the DNB, notifying US Customs and Border Protection (CBP) officers who subsequently notify CDC when a person on PHTR attempts to enter the United States at any port of entry (i.e., seaport, airport, or land border) (*7*). Federal PHTRs are typically not applied to domestic travel on trains, buses, or ships because the mechanism for verifying travelers on these conveyances is different than that of the robust, existing system for commercial air travel and international travel across US borders.

Federal PHTR can be considered for any persons with a suspected or confirmed disease of public health interest or a HRE to a communicable disease that poses a public health threat should the person become symptomatic during travel (*5*). Before the Ebola virus disease outbreak in 2014, PHTRs had only been used for persons with suspected or confirmed infectious pulmonary tuberculosis (99%) or confirmed measles (*6*) and not for persons at risk for development of a disease of public health interest. Under the revised criteria for federal PHTRs, and in conjunction with the Monitoring and Movement Guidance in place during the 2014–2016 Ebola epidemic (*1*), persons with HRE to Ebola virus were eligible for federal PHTR (*3*).

In addition, persons with HREs to other communicable diseases that posed a public health threat were also eligible for DNB placement. Thus, CDC considered and applied PHTR to persons with HREs to Lassa fever virus and Middle East respiratory syndrome coronavirus (MERS-CoV). These contacts were monitored by occupational health or local or state health departments. Travel restrictions were not considered for contacts of 2 patients with cases of infection with MERS-CoV imported into the United States in 2014 (*8*). Guidance for use of controlled movement for an exposure to MERS-CoV, including use of federal PHTR, has been published (*9*). To illustrate how travel restrictions might protect the health of the traveling public and contribute to enhanced global health security, we describe persons with HREs to a communicable disease of public health interest who were eligible for and placed on PHTR during 2014–2016.

## Methods

CDC maintains case records for persons for whom federal PHTRs are requested in its Quarantine Activity Reporting System, a secure, restricted-access database (*10*). Demographic, clinical, and exposure information is obtained from the requesting agency, typically a local or state health department, as well as evidence that the criteria for implementing and removing PHTR are met and the dates and times of major events leading to placement or removal of federal PHTR. We identified all persons placed on federal PHTRs because of HREs to any communicable disease of public health concern during a 3-year period (2014–2016); persons whose travel was restricted because of a confirmed or suspected communicable disease have been reported elsewhere (*6**,**7*) and were excluded from this analysis.

For all identified persons, we examined demographics including sex, age, and location at time of PHTR placement (i.e., within or outside the United States). We determined the circumstances of the exposure (high-risk or close contact) and the type of contact the person had with the case-patient with the communicable disease (i.e., healthcare, household, or community exposure). In addition, we described the circumstances under which persons were removed, either related to the disease-specific incubation periods or a revised exposure risk classification based on reassessment or a change in guidance, and the number of days spent under PHTR. This record review and analysis was determined by CDC to be Public Health Practice: Non-Research and therefore not subject to review by the CDC Institutional Review Board.

## Results

In the 3-year cohort time frame, all restrictions for persons exposed to a communicable disease of public health concern were implemented during a 1-year period (August 2014–July 2015); a total of 164 persons were considered eligible for federal PHTR as a result of exposure to Ebola virus, Lassa fever virus, or MERS-CoV. Exposures to Ebola virus and MERS-CoV were related to an ongoing epidemic of those diseases. Of persons eligible, 160 (98%) were placed under PHTR: 142 (89%) persons were exposed to Ebola virus in the United States or West Africa, 16 (10%) were contacts of a confirmed case-patient with Lassa fever imported into the United States, and 2 (1%) were exposed to MERS-CoV during an outbreak in South Korea (Table 2). Four (3%) persons were not placed under PHTR because of imminent ending of the monitoring period for the patient or insufficient identifying information needed for placement on PHTR. Most (154, 96%) persons were located in the United States at the time of placement. Median age was 38 years (range 5 months–72 years); 49 (31%) were male, and 84 (52%) were female. Sex was not reported for 27 (17%) contacts.

**Table 2:** Characteristics of persons placed on federal public health travel restrictions because of higher-risk exposure to a communicable disease or pathogen of public health concern, January 2014–December 2016*

Characteristic	**Ebola**	**Lassa fever**	**MERS-CoV**	**Total**
No. contacts identified	142	16	2	160
Median age, y (range)	38 (0–71)	39 (1–69)	51 (39–72)	38 (0–72)
Sex				
M	44	4	1	49 (31)
F	72	11	1	84 952)
Not reported	26	1	0	27 (17)
Location at time of placement				
United States	138	16	0	154 (96)
Outside continental United States	4	0	2	6 (4)
*Values are no. (%) persons except as indicated. MERS-CoV, Middle East respiratory syndrome coronavirus.				

Of those placed under PHTR, 136 (85%) were removed after completion of the incubation period (14 days for infection with MERS-CoV, 21 for Ebola and Lassa fever) on the basis of the last day of exposure and after confirmation that they remained asymptomatic. Another 20 (13%) were removed because of a revised exposure risk classification after a change in guidance, and 4 (2%) were removed because of a revised exposure risk classification based on reassessment. Ebola contacts were on PHTR for an average of 12 days, MERS-CoV contacts 9 days, and the Lassa fever contacts 13.5 days. None of the persons on PHTR attempted to travel into, out of, or within the United States.

### Persons Exposed to Ebola in the United States

Most (128, 88%) persons eligible for PHTR for an Ebola exposure were exposed to 1 of 4 cases of Ebola virus disease identified in the United States: 2 imported cases and 2 locally acquired cases (*11**–**13*) (Table 3). During October 7–November 2014, a total of 124 (97%) contacts were placed on PHTR (Figure).

**Table 3:** Types of contacts, by risk level, identified for federal travel restrictions because of exposure to 4 case-patients given a diagnosis of Ebola in the United States, October 7–November 14, 2014*

**Risk level**	**Case-patient 1**		**Case-patient 2**		**Case-patient 3**		**Case-patient 4**		**Total**
	High risk	Close contact	High risk	Close contact	High risk	Close contact	High risk	Close contact	
**No. contacts identified**	52	1	24	0	14	34	3	0	128
**Household contact**	0	0	0	0	0	0	1	0	1
**Healthcare exposure**	51	0	23	0	8	0	0	0	82
**Community contact†**	1	1	1	0	6	34	2	0	45
**Contacts placed on travel restrictions‡**	49	1	24	0	13	34	3	0	124

*High risk was defined as being within ≈3 feet (1 m) of a person with symptomatic Ebola for a prolonged period while not using appropriate personal protective equipment. †Includes 20 contacts with persons on airplanes. ‡Two healthcare workers and 1 community contact with an exposure to case-patient 1 were not placed on travel restrictions because their 21-d incubation periods were scheduled to end 1 day after they were to be placed under travel restrictions. One community contact exposed to case-patient 3 was not placed on travel restrictions because of insufficient biographical data needed for placement.

**Figure:**
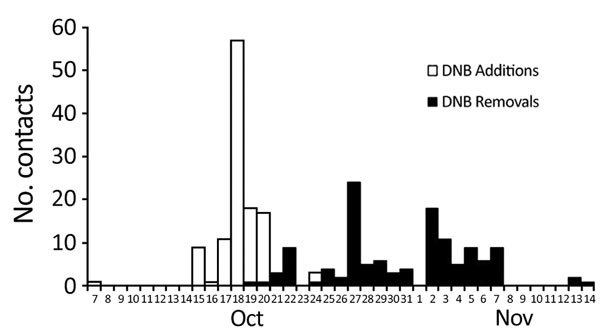
Timeline of federal public health travel restriction actions for 124 contacts of US case-patients with Ebola, October 7–November 14, 2014. DNB, do not board.

The state health department (SHD) of jurisdiction identified 53 contacts for the first Ebola case-patient, who had traveled from Liberia to the United States before becoming symptomatic. Controlled movement was indicated for all contacts, and 50 (94%) were subsequently placed on PHTR; 3 (6%) contacts were not placed on PHTR because their 21-day monitoring period was scheduled to end 1 day after they were identified as needing travel restrictions. Of the 50 contacts who were placed on PHTR, 49 (98%) were healthcare workers who were assessed as high-risk contacts because of an unidentified breach in infection control in the healthcare facility where the first case-patient was treated. One community contact was considered to have had close contact with the case-patient. This contact was placed on PHTR because the person had imminent travel plans but could not be located, and it was unknown whether the person was symptomatic. None of the 50 contacts showed development of symptoms of Ebola.

Two healthcare workers who provided care to the first case-patient became the second and third confirmed Ebola case-patients in the United States (*11**,**12*). Two SHDs identified 72 contacts who were eligible for PHTR because of their potential exposure to Ebola: 24 contacts of the second case-patient and 48 contacts of the third case-patient. A total of 71 (99%) persons were placed under PHTR; 1 person was not placed because of insufficient identifying information.

Of persons placed on PHTR, 37 (52%) were high-risk contacts and 34 (48%) were identified as having close contact. Among high-risk contacts, 31 (84%) were healthcare workers who had provided care to the second or third case-patients and 6 (16%) were community contacts. Of the 34 contacts initially identified as having close contact, 24 (71%) were removed from PHTR within 1 h after it was determined that their exposure risk classification had changed. Of these 24 contacts, 4 were reclassified after further epidemiologic assessment, and 20 were reclassified after revision of the risk classification guidelines (*14*). Of the contacts for the second and third case-patients who remained on PHTR for the duration of their incubation periods (47, 66%), none showed development of symptoms of Ebola.

In October 2014, a fourth case of Ebola diagnosed in the United States was reported in a healthcare worker who had returned from West Africa (*13*); this case was not related to the other cases. The health department identified 3 high-risk contacts: 2 in the community and 1 in the household of the case-patient. All contacts were placed on PHTR and remained asymptomatic.

### Persons Exposed to Ebola Outside the United States

Four contacts located outside the United States were identified and placed under PHTR because of exposures to Ebola cases in West Africa. Two contacts were household contacts of a deceased patient with Ebola in West Africa; the contacts had confirmed commercial travel scheduled to the United States during their 21-day incubation periods. Both contacts were removed from PHTR immediately after their incubation period, and we confirmed that neither contact became symptomatic. In addition, 2 HRE contacts were reported to CDC by foreign ministries of health (MOHs) and placed under PHTR because they reportedly had planned commercial air travel before completing the monitoring period. None of these contacts attempted to travel to the United States while on PHTR.

During December 2014–April 2015, a total of 14 persons were identified as having had a high-risk exposure to Ebola while working in and around an Ebola treatment center in West Africa that reported unsafe infection control practices. Upon their return to the United States by chartered flight, these persons were subjected to controlled movement and placed under PHTR. These persons were monitored by state public health officials and all remained asymptomatic.

### Persons Exposed to Lassa Fever or MERS-CoV

CDC used federal PHTR during May–July 2015 for high-risk contacts of a person exposed to an imported case of Lassa fever and persons exposed during an international outbreak of infection with MERS-CoV. In May 2015, a person who had traveled from West Africa was confirmed to have Lassa fever, and 16 persons were identified as being high-risk contacts: 6 (37%) household contacts, 7 (44%) community contacts, and 3 (19%) healthcare providers. These persons were closely monitored by the SHD and removed from PHTR after completing their incubation periods. In July 2015, in response to the MERS-CoV disease outbreak in South Korea, CDC identified 2 contacts with confirmed commercial air travel involving the United States; both contacts were considered community contacts of patients infected with MERS-CoV and were placed on PHTR. One person was identified in a US territory, monitored until completion of the incubation period, and removed from PHTR. The second person was placed under quarantine in South Korea until completing the incubation period, at which time this person was removed from PHTR and able to travel back to the United States.

## Discussion

CDC recommended controlled movement for persons with HRE to these high-consequence diseases because of the risk for a person becoming symptomatic and exposing others during commercial travel; federal travel restriction tools were used to support recommendations outlined in published movement and monitoring guidance and in the Federal Register (*1**,**3**,**9*). These PHTRs aligned with recommendations of the International Health Regulations 2005 in response to specific public health risks, as well as with the World Health Organization Emergency Committee guidelines regarding travel restrictions for persons with Ebola and contacts (*15**,**16*). No person in this cohort attempted commercial air travel while under PHTR, suggesting that the use of federal travel restriction tools might reinforce the need for adhering to public health recommendations.

Federal PHTR reduced the risk for disease transmission among the traveling public even if any of the restricted persons chose not to comply with public health recommendations and had attempted commercial air travel. Most persons with HREs were located in the United States and under direct active monitoring along with community-level movement restrictions imposed by state authorities. All public health actions regarding travel restrictions were coordinated between state/local authorities and CDC.

Federal PHTR provided support and assurance for SHDs, especially during the 2014–2016 Ebola epidemic, when SHDs were monitoring several thousand persons with various risk classifications for symptoms, in addition to those persons with HREs who were placed on PHTR (*17*). Because most persons in the data cohort were located in the United States, their exposure risk assessments were completed by SHDs, who made the request for PHTR for those persons with HREs. Foreign MOHs or CDC assessed the risk for persons abroad who were planning to travel. Exposure risk classifications were developed with CDC subject matter experts and aligned with published disease-specific movement and monitoring guidance (*2*). CDC continues to evaluate travel restriction criteria as it relates to persons with HREs and disease-specific exposure risk classifications and might refine them as needed during future outbreaks.

Consistent communication and strong collaboration with partners was critical for successfully implementing travel restrictions. The nature and volume of persons placed on PHTR in compressed timeframes during the Ebola outbreak was unprecedented and required close collaboration between local, state, federal, and international partners, as well as the travel industry. SHDs or foreign MOHs were responsible for obtaining biographical data for contacts, determining date of exposure and exposure risk level, and then requesting PHTR placement if a contact was placed under controlled movement. SHDs and MOHs worked with CDC to track dates for removal from PHTR on the basis of incubation periods of contacts to ensure PHTR were removed as soon as the incubation period of the person had been completed. CDC worked closely with the US Department of Homeland Security, using standard processes of internal and external approvals (*6**,**18*), to promptly implement and remove PHTR for a large number of persons over a short timeframe.

CDC notified all US-based persons of their placement on and removal from PHTRs; notifications to contacts outside the United States were provided to in-country public health officials who then informed the persons. All persons were compliant with public health recommendations for controlled movement, and none contested their travel restrictions. States assisted persons on PHTRs who were housed at locations near a healthcare facility during their incubation periods. US Department of State assistance was made available to those US citizens placed under PHTRs while located overseas.

In addition, CDC worked closely with the airline industry to minimize the burden for those persons who had travel planned during their incubation period and issued formal requests for airline change fee waivers. The established relationship between CDC and the airline industry was critical to the successful waiver of change fees for persons. US-based airlines generally do not have established criteria for denying boarding for ill passengers and follow CDC recommendations for restricting travel of persons as it protects the health of other passengers traveling on their aircraft.

Challenges in implementing and removing PHTRs for this data cohort were related to the large number of urgent requests for PHTRs over short periods during outbreaks. Implementing and removing PHTRs are detailed administrative processes requiring extensive resources to coordinate an all-hours response to a large number of urgent requests for PHTRs. After the Ebola outbreak, CDC trained surge staff in administrative process for implementing and removing PHTRs as a means to supplement personnel resources during future outbreaks that generate a high volume of urgent requests for PHTRs.

Under the revised criteria for federal PHTRs (*3*), and in conjunction with disease-specific Monitoring and Movement Guidance, such as that published for Ebola virus and MERS-CoV (*1**,**9*), PHTRs are valuable tools for state and local officials, as well as foreign MOHs, during outbreaks of communicable diseases of public health concern. PHTRs reinforce recommended controlled movement of persons with HREs to communicable diseases, even if these persons refuse to comply with public health monitoring and recommendations to postpone commercial travel. PHTRs can enhance global health security by providing a mechanism to mitigate international importation, transmission, and spread of highly communicable diseases during epidemics of high consequence or emerging infectious diseases.
